# Supplementary material for: Uric Acid Reacts with Peroxidasin, Decreases Collagen IV Crosslink, Impairs Human Endothelial Cell Migration and Adhesion
Source: Antioxidants (Basel). 2022 Jun 4;11(6):1117. doi: 10.3390/antiox11061117 (PMC9220231; doi:10.3390/antiox11061117)
Supplement: Supplementary file 1 [file antioxidants-11-01117-s001.zip › antioxidants-1732317-supplementary.pdf]

Supplementary Material

Uric acid reacts with peroxidasin, decreases collagen IV crosslink, and impairs human endothelial cell migration and adhesion

Bianca Dempsey*, Litiele Cezar Cruz*, Marcela Mineiro, Railmara Pereira da Silva, Flavia Carla Meotti#

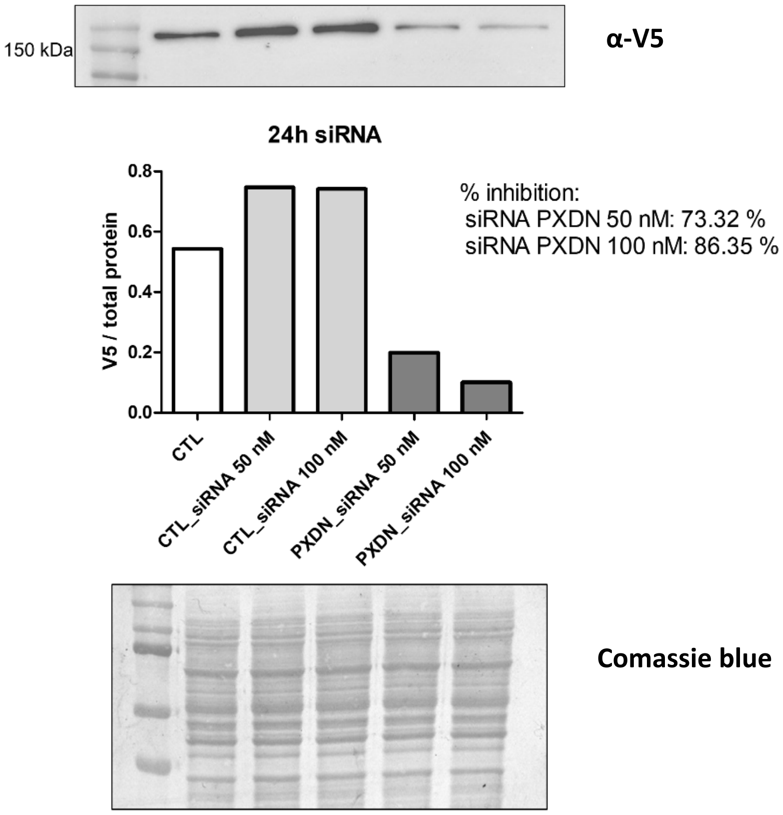

Figure S1: PXDN expression after siRNA PXDN treatment in HEK293/PXDN cells. Cells were treated with 50 or 100 nM siRNA (CTL: scramble siRNA or PXDN) for 24 hours. HEK293/PXDN lysate (20 μg) was separated by a 10% SDS-PAGE and PXDN identified by immunoblotting of Anti-V5 tag antibody. The control (CTL, white bar) group represents cells without lipofectamine treatment. V5 antibody signal was normalized by total protein content (membrane stained with comassie blue).

Table S1: List of PXDN unique peptides found in HUVEC samples by DDA proteomics.

Peptides	Mass	ECM	Secretome (1h30 min)	Secretome (24h)	SDS-PAGE 1h30 min (100-250 kDa)	SDS-PAGE 24h (100-250 kDa)
AGEIFER	820.4079	X				
NEIQSIDR	973.4829	X				
TQEVTLR	845.4607	X				
EIQPGAFR	916.4767				X	X
IATELLK	786.4851			X	X	
IVNEGGIDPLLR	1294.7250				X	
DGVQVTESGK	1018.4931		X	X		
DHGIPPYHDYR	1368.6211		X			
GDRTPLPVDPR	1221.6466				X	
GLASLEQLYLHFNQIETLDPDSFQHLPK	3253.6295	X				
IPSGAFEDLENLK	1431.7246				X	
LFLHNNR	912.4930		X			
LFSMAHTVALDLAAINIQR	2099.1197			X		
LLKSVYENGFNTPR	1636.8573					X
LYNGHALPMPR	1268.6336				X	
NGDPFVATSIVEAIATVDR	1974.9898			X		
NVAGEVKTQEVTLR	1542.8366	X			X	X
SPNDLLALFR	1144.6241				X	
SSPVCGSGMTSLLMNSVYPR	2173.9806		X	X		
SVYENGFNTPR	1282.5942			X	X	X
TQEVTLR	8454.6068	X				
VPSQLLNTELTER	1498.7990				X	

Table S2: List of laminin unique peptides found in HUVEC and HEK293 samples by DDA proteomics

Protein IDs	Majority protein IDs	Peptide counts (all)	Peptide counts (razor+unique)	Peptide counts (unique)	Protein names	Gene names
O15230	O15230	2	2	2	Laminin subunit alpha-5	LAMA5
P07942	P07942	6	6	6	Laminin subunit beta-1	LAMB1
P11047	P11047	14	14	14	Laminin subunit gamma-1	LAMC1
P25391	P25391	2	2	2	Laminin subunit alpha-1	LAMA1

Fasta headers	Number of proteins	Peptides	Razor + unique peptides	Unique peptides
sp|O15230|LAMA5_HUMAN Laminin subunit alpha-5 OS=Homo sapiens OX=9606 GN=LAMA5 PE=1 SV=8	1	2	2	2
sp|P07942|LAMB1_HUMAN Laminin subunit beta-1 OS=Homo sapiens OX=9606 GN=LAMB1 PE=1 SV=2	1	6	6	6
sp|P11047|LAMC1_HUMAN Laminin subunit gamma-1 OS=Homo sapiens OX=9606 GN=LAMC1 PE=1 SV=3	1	14	14	14
sp|P25391|LAMA1_HUMAN Laminin subunit alpha-1 OS=Homo sapiens OX=9606 GN=LAMA1 PE=1 SV=2	1	2	2	2

Peptides ECM_HEK_PXDN_1	Peptides ECM_HEK_PXDN_2	Peptides ECM_HEK_PXDN_3	Peptides ECM_HEK_WT_1	Peptides ECM_HEK_WT_2	Peptides ECM_HEK_WT_3	Peptides ECM_HUVEC_1	Peptides ECM_HUVEC_2	Peptides ECM_HUVEC_3
0	0	0	0	1	1	0	0	0
5	5	3	2	3	2	0	0	0
13	11	10	8	7	8	2	1	1
2	2	1	0	0	0	0	0	0

Razor + unique peptides ECM_HEK_PXDN_1	Razor + unique peptides ECM_HEK_PXDN_2	Razor + unique peptides ECM_HEK_PXDN_3	Razor + unique peptides ECM_HEK_WT_1	Razor + unique peptides ECM_HEK_WT_2	Razor + unique peptides ECM_HEK_WT_3	Razor + unique peptides ECM_HUVEC_1	Razor + unique peptides ECM_HUVEC_2	Razor + unique peptides ECM_HUVEC_3
0	0	0	0	1	1	0	0	0
5	5	3	2	3	2	0	0	0
13	11	10	8	7	8	2	1	1
2	2	1	0	0	0	0	0	0

Unique peptides ECM_HEK_PXDN_1	Unique peptides ECM_HEK_PXDN_2	Unique peptides ECM_HEK_PXDN_3	Unique peptides ECM_HEK_WT_1	Unique peptides ECM_HEK_WT_2	Unique peptides ECM_HEK_WT_3	Unique peptides ECM_HUVEC_1	Unique peptides ECM_HUVEC_2	Unique peptides ECM_HUVEC_3
0	0	0	0	1	1	0	0	0
5	5	3	2	3	2	0	0	0
13	11	10	8	7	8	2	1	1
2	2	1	0	0	0	0	0	0

Sequence coverage [%]	Unique + razor sequence coverage [%]	Unique sequence coverage [%]	Mol. weight [kDa]	Sequence length	Sequence lengths	Fraction average	Fraction 1	Q-value	Score
0.8	0.8	0.8	399.73	3695	3695	1	2	0.0011648	11,422
3.4	3.4	3.4	198.04	1786	1786	1	20	0	35,057
12.6	12.6	12.6	177.6	1609	1609	1	70	0	100.05
0.8	0.8	0.8	337.08	3075	3075	1	5	0.00065189	13,998

Identification type ECM_HEK_PXDN_1	Identification type ECM_HEK_PXDN_2	Identification type ECM_HEK_PXDN_3	Identification type ECM_HEK_WT_1	Identification type ECM_HEK_WT_2	Identification type ECM_HEK_WT_3	Identification type ECM_HUVEC_1	Identification type ECM_HUVEC_2	Identification type ECM_HUVEC_3
				By MS/MS	By MS/MS			
By MS/MS	By MS/MS	By MS/MS	By MS/MS	By MS/MS	By MS/MS			
By MS/MS	By MS/MS	By MS/MS	By MS/MS	By MS/MS	By MS/MS	By MS/MS	By matching	By MS/MS
By MS/MS	By MS/MS	By MS/MS						

Sequence coverage ECM_HEK_PXDN_1 [%]	Sequence coverage ECM_HEK_PXDN_2 [%]	Sequence coverage ECM_HEK_PXDN_3 [%]	Sequence coverage ECM_HEK_WT_1 [%]	Sequence coverage ECM_HEK_WT_2 [%]	Sequence coverage ECM_HEK_WT_3 [%]	Sequence coverage ECM_HUVEC_1 [%]	Sequence coverage ECM_HUVEC_2 [%]	Sequence coverage ECM_HUVEC_3 [%]
0	0	0	0	0.4	0.4	0	0	0
3.4	2.7	2	1.6	1.9	1.6	0	0	0
11.7	10	9.4	7.5	6.5	7.2	2.4	1.4	1.1
0.8	0.8	0.4	0	0	0	0	0	0

Intensity	Intensity ECM_HEK_PXDN_1	Intensity ECM_HEK_PXDN_2	Intensity ECM_HEK_PXDN_3	Intensity ECM_HEK_WT_1	Intensity ECM_HEK_WT_2	Intensity ECM_HEK_WT_3	Intensity ECM_HUVEC_1	Intensity ECM_HUVEC_2	Intensity ECM_HUVEC_3
1769900	0	0	0	0	0	1769900	0	0	0
17133000	4457400	4839900	1551900	1915000	2485600	1883400	0	0	0
122390000	37138000	17972000	9277700	11815000	28266000	14950000	1679700	461040	829090
3684500	1433700	1686400	564380	0	0	0	0	0	0

LFQ intensity ECM_HEK_PXDN_1	LFQ intensity ECM_HEK_PXDN_2	LFQ intensity ECM_HEK_PXDN_3	LFQ intensity ECM_HEK_1	LFQ intensity ECM_HEK_2	LFQ intensity ECM_HEK_3	LFQ intensity ECM_HUVEC_1	LFQ intensity ECM_HUVEC_2	LFQ intensity ECM_HUVEC_3
0	0	0	0	0	0	0	0	0
1374200	1913100	1107300	962910	956600	0	0	0	0
11041000	11732000	13411000	8065100	6204500	8677400	3297400	0	0
1055600	1278000	0	0	0	0	0	0	0

MS/MS count ECM_HEK_PXDN_1	MS/MS count ECM_HEK_PXDN_2	MS/MS count ECM_HEK_PXDN_3	MS/MS count ECM_HEK_WT_1	MS/MS count ECM_HEK_WT_2	MS/MS count ECM_HEK_WT_3	MS/MS count ECM_HUVEC_1	MS/MS count ECM_HUVEC_2	MS/MS count ECM_HUVEC_3	MS/MS count
0	0	0	0	1	1	0	0	0	2
3	3	1	1	0	0	0	0	0	8
8	8	9	6	5	5	1	0	1	43
2	1	1	0	0	0	0	0	0	4

Peptide sequences
GALDQLCGAGGLCR;SVEVHGAVGASGCPAA
FGYYGDALQQDCR;FGYYGDALQQDCRK;IPSWTGAGFVR;LQLLKDLER;RKAEMLQNEAK;YSDIEPSTEGEVIFR
AFDITYVR;CIYNTAGFYCDR;EAQQALGSAAADATEAK;LCQCSDNIDPNAVGNCNR;LKDYEDLREDMR;LNTFGDEVFNDPK;NTIEETGNLAEQAR;QLQEAEKELK;RCELCDDGYFGDPLGR;SQECYFDPELYR;STGHGGHCTNCQDNTDGAHCER;SYYYAISDFAVGGR;TFAEVTDLDNEVNNMLK;TGQCECQPGITGQHCER
KLPGIALELSELR;TPVTLGSDQPLLR

Only identified by site	Reverse	Potential contaminant	id	Peptide IDs
			90	4156;13671
			295	3663;3664;6488;9157;12010;16851
			338	425;1732;2571;7892;8517;9011;11050;11547;11856;13313;13584;13807;14014;14112
			455	7334;14604

Peptide is razor	Mod. peptide IDs
True;True	4185;13769
True;True;True;True;True;True	3683;3684;6528;9215;12095;16970
True;True;True;True;True;True;True;True;True;True;True;True;True;True	426;1744;2585;7941;8571;9065;11133;11632;11941;13407;13681;13905;14114;14212
True;True	7380;14705

Evidence IDs	MS/MS IDs
29549;98191	23709;77896
25706;25707;25708;25709;25710;25711;25712;46735;46736;66567;66568;66569;86550;86551;86552;86553;121738;121739;121740;121741	20523;20524;20525;20526;37546;53136;68529;96947;96948
2734;2735;2736;2737;2738;12458;12459;18366;18367;18368;18369;18370;57864;57865;57866;62175;62176;65567;65568;65569;65570;65571;65572;65573;80047;80048;83404;83405;85468;85469;85470;85471;95581;95582;95583;95584;95585;95586;95587;97589;97590;97591;97592;97593;97594;97595;97596;97597;97598;97599;99082;99083;99084;99085;99086;99087;99088;99089;99090;99091;100624;100625;101256;101257;101258;101259;101260;101261;101262;101263	2230;2231;10013;14750;14751;46324;46325;46326;49728;52398;52399;63568;63569;66135;67739;75707;75708;75709;75710;75711;77417;77418;77419;77420;77421;77422;78586;78587;78588;78589;78590;78591;78592;78593;78594;79824;79825;79826;80330;80331;80332;80333;80334;80335
53714;53715;104816;104817;104818	43206;83226;83227;83228

Best MS/MS	Oxidation (M) site IDs	Oxidation (M) site positions	Taxonomy IDs
23709;77896			-1
20523;20524;37546;53136;68529;96947			-1
2230;10013;14751;46324;49728;52399;63569;66135;67739;75709;77420;78594;79825;80334			-1
43206;83227			-1
